# Correlation of Macular Focal Electroretinogram with Ellipsoid Zone Extension in Stargardt Disease

**DOI:** 10.1155/2017/3643495

**Published:** 2017-08-20

**Authors:** Edoardo Abed, Giorgio Placidi, Luigi Calandriello, Marco Piccardi, Francesca Campagna, Matteo Bertelli, Angelo Maria Minnella, Maria Cristina Savastano, Benedetto Falsini

**Affiliations:** Ophthalmology Department, Università Cattolica del Sacro Cuore, Largo Agostino Gemelli 8, 00168 Rome, Italy

## Abstract

Stargardt disease (STGD1) is the most common cause of inherited juvenile macular degeneration. This disease is characterized by a progressive accumulation of lipofuscin in the outer retina and subsequent loss of photoreceptors and retinal pigment epithelium. The aim of this study was to evaluate the relationship between cone photoreceptor function and structure in STGD1. Macular function was assessed by visual acuity measurement and focal electroretinogram (FERG) recording while spectral domain optical coherence tomography (SD-OCT) imaging was performed to evaluate the integrity of photoreceptors. FERG amplitude was significantly reduced in patients with Stargardt disease (*p* < 0.0001). The amplitude of FERG showed a negative relationship with interruption of ellipsoid zone (EZ) (*R*^2^ = 0.54, *p* < 0.0001) and a positive correlation with average macular thickness (AMT). Conversely, visual acuity was only weakly correlated with central macular thickness (CMT) (*R*^2^ = 0.12, *p* = 0.04). In conclusion, this study demonstrates that FERG amplitude is a reliable indicator of macular cone function while visual acuity reflects the activity of the foveal region. A precise assessment of macular cone function by FERG recording may be useful to monitor the progression of STGD1 and to select the optimal candidates to include in future clinical trials to treat this disease.

## 1. Introduction

Autosomal recessive Stargardt disease (STGD1) is the most frequent childhood hereditary macular dystrophy, affecting 1 per 10000 individuals [1].

STDGD1 is caused by the mutation of the ATP-binding cassette, subfamily A, member 4 (ABCA4) gene, which encodes a transport protein localized in photoreceptor outer segments [2, 3]. The main role of ABCA4 protein is to remove potentially toxic retinoids, such as N-retinylidene-phosphatidylethanolamine (NRPE) and phosphatidylethanolamide (PE), which originated from the phototransduction process [4].

Mutations of ABCA4 gene determine an increase of NRPE and PE with generation of N-retinylidene-N-retinylethanolamine (A2E), a lipofuscin fluorophore.

The shedding and phagocytosis of photoreceptors outer segments lead to lipofuscin accumulation in the retinal pigment epithelium (RPE) with subsequent RPE apoptosis and photoreceptor degeneration [5].

In the past 20 years, pattern electroretinogram (PERG) has been extensively used to assess macular function in patients with STGD1 [6, 7]. PERG is not recordable in almost all patients with STGD1 even in the case of preserved visual acuity and in the absence of clear signs of macular degeneration at fundus examination. As a consequence, PERG is a valuable tool to establish the diagnosis of STDG1, especially in the earlier phases of the disease. However, for the same reason, PERG may be inappropriate to estimate the degree of macular dysfunction and to assess disease progression. Furthermore, PERG signal reflects the activity of retinal ganglion cells and the inner retina [8] while STDG1 primarily affects the RPE and photoreceptor cells.

Flicker focal electroretinogram (FERG) is a diagnostic tool that selectively assesses macular cone photoreceptor and bipolar cell activity [9]. In previous clinical studies, this technique showed good test-retest repeatability, allowing a long term follow-up of macular dysfunction in retinitis pigmentosa [10] and cone-rod dystrophy [11]. Furthermore, it has been demonstrated that, in cone-rod dystrophies, FERG amplitude decline may anticipate visual acuity loss of several years [12].

Spectral domain optical coherence tomography (SD-OCT) is a noninvasive imaging technique that provides information about the morphology of all distinct retinal layers and enables reliable and repeatable measurements of macular thickness. In patients with STDG1, SD-OCT has allowed clinicians to accurately visualize the extent and the degree of the degeneration of both inner and outer retinal layers [13–15]. More specifically, ellipsoid zone extension can be considered an important imaging parameter that allows clinicians to evaluate to the extent of damage of macular photoreceptors [16].

The aim of our study was to assess macular cone function by FERG recording in STDG1 and to explore the correlation between photoreceptor function and structure as determined by FERG and SD-OCT, respectively.

## 2. Methods

Patients with STDG1 evaluated at the Department of Ophthalmology of the Catholic University of Rome between June 2012 and June 2015 were retrospectively enrolled. The diagnosis of STDG1 was clinically established and then confirmed, in all patients, by genetic testing with next generation sequencing (NGS) technology. The examination protocol included best-corrected visual acuity (BCVA) measurement with Snellen charts, slit-lamp biomicroscopy, indirect ophthalmoscopy, spectral domain optical coherence tomography (SD-OCT), and FERG recording. Twenty age-matched healthy patients were also enrolled and served as controls for FERG amplitude.

The study followed the tenets of the Declaration of Helsinki and was approved by the Ethics Committee of the Catholic University of Sacred Heart of Rome. All patients signed a written informed consent before the enrollment.

### 2.1. Imaging

SD-OCT scans were performed using the Cirrus OCT (Carl Zeiss Meditec Inc., Dublin, California, USA). The examination protocol consisted of a 6 × 6 mm macular cube, centered on the fovea, composed of 128 horizontal b-scans of 512 a-scans each. Retinal thickness values were automatically calculated by Cirrus OCT software for each of the nine areas corresponding to the Early Treatment Diabetic Retinopathy Study Research Group (ETDRS) [17, 18]. Both the average macular thickness (AMT) and the central macular thickness (CMT) were recorded. The AMT and CMT correspond to the mean retinal thickness in the circular zones of 6 and 1 mm diameter, respectively, centered in the fovea.

Cirrus software was also used to navigate the acquired macular cube in order to identify the horizontal b-scan with the largest interruption of photoreceptor ellipsoid zone (EZ). The maximum extent of EZ interruption was then manually measured by two independent examiners using the calipers of Cirrus software. In the case of foveal sparing, the amplitude of EZ zone interruption nasally and temporally to the fovea was measured and summed.

### 2.2. Focal Electroretinography

FERG recording was performed in accordance to a previously described technique [9–19]. Before examination, pupils were pharmacologically dilated with tropicamide 1% eye drops to at least 8 mm diameter. FERGs were recorded monocularly with an Ag-AgCl electrode taped on the skin over the lower eyelid. A similar electrode was placed over the eyelid of the contralateral patched eye and was used as reference.

Stimuli consisted of flickering uniform fields generated by an array of 8 red light-emitting diodes covering a 18° diameter with a mean luminance of 80 cd/m2 and a temporal frequency of 41 Hz. The dominant wavelength of the stimulus was 630 nm. The flickering stimulus was sinusoidally driven by a custom-made digital frequency generator and presented on the rear of a modified Ganzfeld bowl (Primus; LACE Elettronica, Pisa, Italy) illuminated at the same mean luminance as the stimulus.

A diffusing filter was placed in front of the LED array in order to make it appear as a circle of uniform red light. A steady DC signal maintained the mean luminance of the stimulus. A small square marker was placed in the center of the Ganzfeld bowl to allow the investigator maintaining steady fixation on the foveal region. The examined patients were placed at 30 cm distance from the stimulus.

FERG signals were amplified, filtered (band pass filter between 1 and 250 Hz), and averaged (12-bit resolution, 2 kHz sampling rate, 1600 repetitions in 8 blocks). Signals exceeding the threshold voltage (25 mV) were rejected to minimize noise coming from blinks or eye movements. After the recording, a Fourier discrete analysis was performed to isolate the FERG's first harmonic (1F), and its peak-to-peak amplitude was measured. Averaging and Fourier analysis were also performed on signals sampled asynchronously at 1.1 times the temporal frequency of the stimulus, to estimate the background noise at the fundamental component. Under these conditions, the FERGs recorded were above the noise level (noise amplitude <0.08 mV in all cases) and sufficiently reliable (the variation coefficient in amplitude was 20%).

### 2.3. Statistical Analysis

Data from the right eye of both patients with STGD1 and controls were used for the analysis to reduce the risk of data redundancy. Two-tailed unpaired *t*-test was used to compare FERG 1F amplitude between the two groups and to compare BCVA and FERG amplitude between STGD1 patients with or without foveal involvement. Analysis of variance and Sidak post hoc test was performed to compare macular functional and anatomical impairment between patients with different mutation severity and patient with early (<17 years) and late (>17 years) age of onset. Receiving operating characteristic (ROC) curves were performed to assess whether FERG alterations may be useful to diagnose STGD1 and to predict photoreceptors loss in these patients.

Pearson's correlation test was used to correlate macular functional and anatomical parameters. A *p* value < 0.05 was considered statistically significant.

## 3. Results

Thirty-three patients (18 males and 15 females) with STGD1 were included in this study. Patients' demographics and molecular and functional characteristics are reported in Table 1, while the results of OCT examination are reported in Table 2.

Mean age at observation was 34.4 ± 16.5 years while mean age of symptoms onset was 15.8 ± 8.9 years. Eight patients had one missense mutation in each allele (24.2%), and 16 patients (48.5%) had two or more missense mutations in at least one allele while the remaining 9 patients (27.3%) had a null mutation in at least one allele. Mean CMT, AMT, and EZ interruption were 141 ± 40, 217 ± 35, and 3911 ± 1423 *μ*m, respectively.

**Figure 1:**
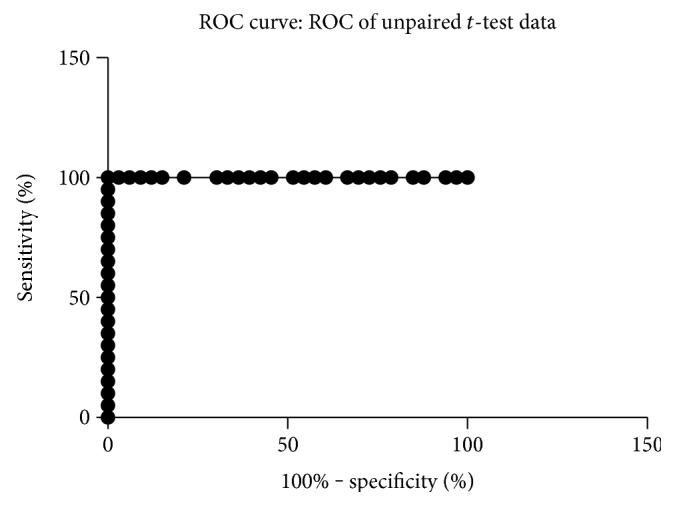
ROC curve showing diagnostic accuracy of FERG 1F amplitude in detecting Stargardt macular dystrophy.

FERG was recordable in all patients of both study groups. FERG 1F amplitude was significantly reduced in patients with STGD1 compared to controls (0.35 ± 0.22 and 1.68 ± 0.27, resp., *p* < 0.0001). The diagnostic accuracy of FERG amplitude to distinguish STGD1 and healthy patients, assessed by ROC analysis, was 100% (area under the curve 1.0). The corresponding ROC curve is shown in Figure 1.

**Figure 2:**
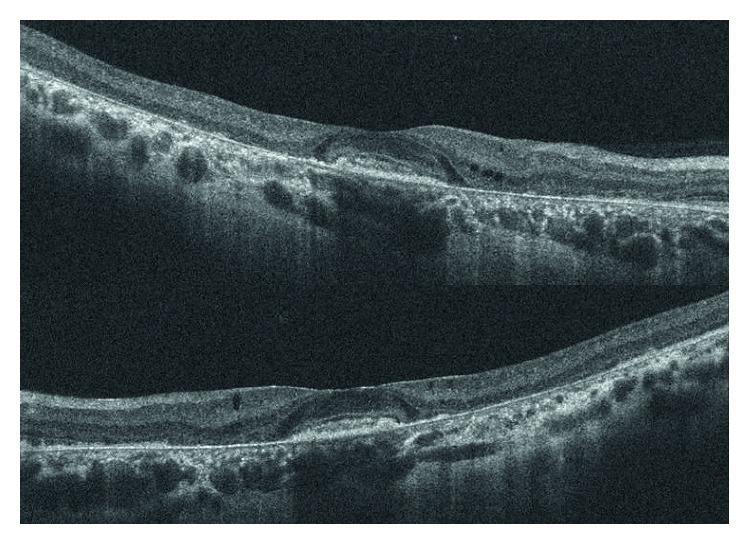
SD-OCT scans of the right and left eye of a patient with significant disruption of macular photoreceptors and foveal sparing. While FERG 1F amplitude was severely reduced, BCVA was 1.0 in both eyes.

Interestingly, while FERG 1F amplitude was significantly reduced (<1 *μ*V) in all patients with STGD1, six patients (18%), due to foveal sparing, had a good visual acuity (≥0.8) in one or both eyes despite a remarkable interruption of EZ (Figure 2). Patients with foveal sparing had significantly better BCVA than those with foveal involvement (0.90 ± 0.05 and 0.13 ± 0.02, resp., *p* < 0.0001) while no differences were noted in FERG 1F amplitude between these two groups of patients (0.20 ± 0.04 and 0.38 ± 0.04, resp., *p* = 0.07).

Correlations between macular functional and structural parameters are summarized in Table 3.

**Figure 3:**
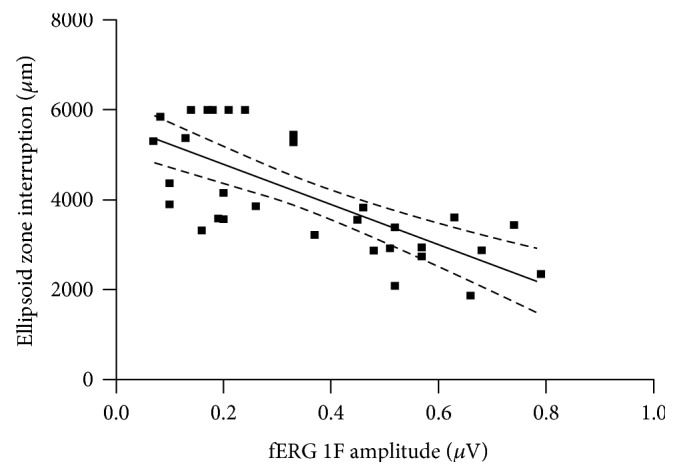
Scatterplot illustrating the significant correlation between FERG first harmonic amplitude and ellipsoid zone interruption.

FERG 1F amplitude was negatively correlated with the extension of EZ interruption (*R*^2^ = 0.54, *p* = 0 < 0001) (Figure 3) and positively correlated with AMT (*R*^2^ = 0.16, *p* = 0.02). Conversely, no correlation was noted between FERG amplitude and CMT (*R*^2^ = 0.003, *p* = 0.76). BCVA did not correlate with anatomical alterations, except a weak negative relationship with CMT (*R*^2^ = 0.12, *p* = 0.04).

Interestingly, no differences were noted regarding FERG 1F amplitude, BCVA, and OCT parameters between patients with early or late onset or with different molecular mutation severity (*p* > 0.05 for all analyses).

## 4. Discussion

The aim of our study was to explore the correlation between functional and anatomical photoreceptor alterations in patients with STDG1, as evaluated by FERG and SD-OCT, respectively. Compared to controls, FERG 1F amplitude was reduced in patients with STDG1 and ROC analysis showed that focal electroretinogram had a diagnostic accuracy of 100% in discriminating healthy and STDG1 patients. These results indicate that, similarly to PERG, FERG recording may be useful to establish the diagnosis of STDG1. Interestingly, despite the significant reduction of macular function, FERG 1F amplitude was recordable in all STDG1 patients, suggesting that this test may be a valuable tool also to assess the deterioration of macular dysfunction in these subjects. A recent study demonstrated that FERG fundamental harmonic alterations anticipate by several years the deterioration of visual acuity in patients affected by cone-rod dystrophies [12]. Longitudinal studies are warranted to determine whether FERG alterations may predict visual acuity decrease in STDG1.

Surprisingly, no significant correlation was noted between BCVA and the entity of photoreceptor loss, determined as the maximal linear EZ interruption. This finding is in contrast with a previous observational case series of 14 patients by Ergun et al. [13] reporting a negative relationship between the extent of ellipsoid zone disruption and visual acuity.

This apparent discrepancy may be related to a relatively high number of patients with foveal sparing in our study. In these cases, the integrity of photoreceptor layers in the foveal region lead to a preservation of visual acuity despite a substantial loss of EZ in the surrounding retina. This assumption seems to be confirmed by the observation that while no relationship was evident between AMT and BCVA, a weak even if significant positive correlation was found between CMT and visual acuity.

Conversely, FERG 1F amplitude showed a significantly negative correlation with the extent of EZ loss and was markedly altered also in cases with foveal sparing whereas, in these patients, BCVA was largely preserved. These data would suggest that BCVA measurement reflects only roughly the function of the foveal region, while FERG signals evocated by an 18° flickering stimulus may provide an objective and reliable quantification of macular cone function.

The exact quantification of macular cone residual activity may play an important role for the optimal enrollment of patients in future clinical trials.

In particular, this assessment may be critical for therapies aimed at preservation or restoration of photoreceptor function such as nutritional treatments [20, 21], growth factor administration [22–25], and gene augmentation [26, 27].

Hence, these findings suggest that FERG recording may be a useful adjunct tool to increase the accuracy of patient's selection in promising future clinical trials.

Interestingly, even if FERG fundamental harmonic amplitude was also correlated with AMT, this correlation was notably weaker.

Our study failed to demonstrate a correlation between early onset and disease severity and the decay of retinal function in patients with STDG1. This finding is apparently in contrast with the results of a previous study by Fujinami et al. [28], showing that childhood onset is associated with a more severe alteration of retinal function. However, that study found only an increased proportion of patients with diffuse impairment of retinal function which was noted in patients with early onset of STDG1 disease compared with adults while visual acuity was not significantly different in the two study groups. This data would suggest that macular function is immediately altered in STDG1 causing an early decay of focal ERG and visual acuity in both adult and childhood onset patients while retinal-wide involvement develops more frequently in patients with early disease onset.

In summary, FERG recording is significantly altered in STDG1 and has an optimal diagnostic accuracy in detecting Stargardt disease. The reduction of FERG 1F amplitude is closely related to the extent of macular photoreceptor disruption. These findings suggest that FERG recording may be a useful tool to monitor the progression of macular dysfunction in Stargardt disease. Furthermore, an accurate estimation of residual cone function will be important to optimize the design and inclusion criteria of future clinical trials to treat STDG1.

## Figures and Tables

**Table 1:** Demographics and clinical characteristics of patients with Stargardt macular dystrophy.

Patient number	Sex	Age	Age at onset	First allele	Second allele	BCVA RE (Snellen decimals)	BCVA LE (Snellen decimals)	FERG 1F RE (*μ*V)	FERG 1F LE (*μ*V)
1	F	22	15	p.R1098C	—	0.1	0.1	0.51	0.28
2	F	33	10	p.V767D	p.R2030X	0.05	0.1	0.17	0.2
3	M	25	15	p.Gly1961Glu, p.Gln1332_cys1339dup	p.Val2026ThrfsX52	0.4	0.4	0.52	0.43
4	F	63	35	p.Gly1961Glu	—	0.15	0.15	0.68	0.38
5	M	58	32	p.Gly991Arg, p.Glu1087Lys	p.Arg1300Gln	0.02	0.02	0.52	0.52
6	M	33	14	p.Asn78Ser	p.Trp880Cys	0.02	0.01	0.21	0.15
7	M	50	24	p.R1098C	—	0.4	0.8	0.52	0.59
8	F	16	9	p.Q2220X	p.R943Q	0.1	0.1	0.08	0.06
9	M	41	16	c.634C>T	c.1497G>C	0.2	0.2	0.74	0.39
10	F	37	21	c.4771G>A	—	0.1	0.1	0.2	0.22
11	M	11	8	c.247_250dup	c.4139C>T	0.8	0.8	0.26	0.34
12	M	41	5	p.His423Arg	p.Arg943Gln	0.1	0.02	0.18	0.15
13	F	18	10	IVS35+2T>C	IVS40+5G>A	0.2	0.2	0.63	0.3
14	M	48	43	c.5714+5G>A	—	1.0	1.0	0.33	0.46
15	F	39	9	p.Arg18Trp	p.Val767Asp	0.05	0.1	0.33	0.35
16	M	15	9	p.Gly1961Glu	p. Ser2255Ile	0.1	0.1	0.79	0.7
17	M	47	19	p.Thr897Ile	—	0.01	0.05	0.1	0.09
18	M	36	31	p.Gln21Ter, p.Arg943Gly	p. Arg212His, p.Gly1961Glu	0.1	0.1	0.37	0.46
19	M	16	13	p.Trp821Arg p.Gly1961Glu	p. Tyr850Cys	0.9	0.9	0.1	0.2
20	F	31	21	p.Thr977Pro	c.IVS40+5G>A	0.2	0.1	0.46	0.15
21	F	22	15	p.Gly1961Glu	c.4709_4711delA	0.1	0.1	0.57	0.45
22	M	48	13	p.Gly1961Glu	p.His1406ProfsX29	0.1	0.1	0.19	0.25
23	M	16	8	p.Val931Met	p.Val931Met	0.1	0.1	0.57	0.4
24	M	42	8	p.Val256splice	p.Trp1479X	0.01	0.005	0.14	0.16
25	M	21	14	IVS13+1g>a	IVS40+5g>40	0.1	0.15	0.2	0.17
26	F	23	17	p.Tyr850Cys, p.Thr959Ala	p.Gly1961Glu	0.3	0.3	0.07	0.09
27	F	56	23	p.Gly1961Glu	p.Gly1961Glu	0.8	0.1	0.16	0.31
28	M	52	20	p.Arg1108His	p.Gly1961Glu	0.1	0.4	0.48	0.93
29	F	31	14	p.P1486L	p.W700X	1.0	1.0	0.13	0.03
30	F	12	8	p.Tyr1400X	p.Val931Met	0.2	0.2	0.45	0.46
31	F	77	13	p.Ala1598Asp	p.Val2062fsX2113	0.1	0.1	0.24	0.33
32	F	11	10	IVS6-1G>T	p.Asn1436Ile	0.2	0.2	0.66	0.95
33	M	44	10	IVS45+1G>A	p.Gly1961Glu	0.1	0.1	0.14	0.15

**Table 2:** OCT data of individual patients with Stargardt disease.

Patient number	EZ interruption RE (*μ*m)	EZ interruption LE (*μ*m)	AMT RE (*μ*m)	AMT LE (*μ*m)	CMT RE (*μ*m)	CMT LE (*μ*m)
1	2922	3665	257	256	121	118
2	6000	6000	289	289	266	273
3	2092	2728	255	257	138	147
4	2872	3428	262	255	155	133
5	3384	3401	237	249	99	80
6	6000	6000	134	145	97	95
7	3398	3145	207	199	186	253
8	5866	5916	154	135	112	98
9	3444	4272	215	198	135	112
10	4156	4453	198	204	121	125
11	3857	3688	185	176	117	113
12	6000	6000	195	187	147	132
13	3600	3445	225	234	145	138
14	5261	4241	224	235	241	251
15	5421	5578	212	203	144	143
16	2353	2535	253	243	166	153
17	3906	4453	248	241	183	157
18	3214	2896	225	231	149	158
19	4370	3999	243	239	160	159
20	3827	4972	212	208	145	134
21	2741	2895	223	243	138	146
22	3585	3352	244	250	104	108
23	2936	3336	243	254	173	171
24	6000	6000	135	147	74	81
25	3575	3936	221	236	125	144
26	5308	6000	212	201	139	127
27	3315	2980	194	197	131	124
28	2867	2100	258	260	100	108
29	5370	5227	218	223	189	209
30	3557	3715	192	185	89	91
31	6000	6000	174	188	95	101
32	1868	1632	227	234	154	161
33	6000	6000	194	187	103	112

**Table 3:** Results of correlation analyses between macular functional and anatomical alterations in patients with Stargardt macular dystrophy.

BCVA	FERG 1 F	*R* ^2^ = 0.04, *p* = 0.27
BCVA	EZ interruption	*R* ^2^ = 0.01, *p* = 0.56
BCVA	CMT	*R* ^2^ = 0.12, *p* = 0.04
BCVA	AMT	*R* ^2^ = 0.0001, *p* = 0.95
FERG 1F	EZ interruption	*R* ^2^ = 0.54, *p* = 0.0001
FERG 1F	CMT	*R* ^2^ = 0.003, *p* = 0.76
FERG 1F	AMT	*R* ^2^ = 0.16, *p* = 0.02
EZ interruption	CMT	*R* ^2^ = 0.14, *p* = 0.03
EZ interruption	AMT	*R* ^2^ = 0.57, *p* < 0.0001
CMT	AMT	*R* ^2^ = 0.30, *p* = 0.001
